# SFRP4^+^IGFBP5^hi^ NKT cells induced neural-like cell differentiation to contribute to adenomyosis pain

**DOI:** 10.3389/fimmu.2022.945504

**Published:** 2022-11-30

**Authors:** Yichen Chen, Jue Zhu, Liang Chen, Yuanyuan Shen, Jing Zhang, Qiming Wang

**Affiliations:** Department of Gynaecology, Ningbo Women & Children's Hospital, Ningbo University, Ningbo, China

**Keywords:** adenomyosis pain, single-cell sequencing, neural-like cells, NKT cells, IGFBP5, SFRP4, neural-like cells

## Abstract

**Background:**

Adenomyosis is an estrogen-dependent gynecological disease. The pathogenesis of chronic pain, the main clinical symptom of adenomyosis, remains undefined. As a combination lymphocyte with both T-cell and natural killer (NK)–cell properties, NK T (NKT) cells play a role in immune defense against numerous diseases and modulate cell differentiation.

**Method:**

This study analyzed the tissue-cell samples from adenomyosis with or without pain by single-cell sequencing.

**Result:**

We found a specific population of secreted frizzled-related protein 4 (SFRP4)^+^NKT cells and a large amount of undifferentiated multipotent stem cells in the adenomyosis pain group. We discovered that a high expression of *IGFBP5* in SFRP4^+^NKT cells could promote the differentiation of multipotent stem cells into neural-like cells *via* the single-cell trajectory. Through verification by the sample, we found that the degree of the expression of the neuronal marker *NEFM* was correlated with the duration of pain in adenomyosis patients. The expression of IGFBP5 was positively correlated with the pain scores of adenomyosis patients.

**Conclusion:**

Collectively, these findings suggest that SFRP4^+^IGFBP5^hi^ NKT cells were capable of converting part of the stem cells into neurogenic cells and inducing adenomyosis pain.

## Introduction

Adenomyosis is a gynecological condition characterized by the presence of endometrial glands and stroma within the myometrial layer of the uterus (1–3). In the last decade, the advancements in imaging techniques have allowed us to detect adenomyosis in young fertile women as well. As a matter of fact, one-third of patients with adenomyosis do not experience symptoms. An estimated 20%–25% of women who undergo assisted reproductive technologies (ARTs) are likely to develop adenomyosis (4), whereas this percentage varies widely among those who have a history of endometriosis (5, 6). According to the data from ultrasound units, there is a prevalence of the sonographic signs of adenomyosis in the general population of 20.9% (7). The clinical symptoms of adenomyosis are diverse, but the majority of patients have painful menstruation and excessive menstrual bleeding, with a minor proportion additionally experiencing pelvic discomfort and dyspareunia (8, 9). There is no doubt that adenomyosis pain has a significant impact on the quality of life of female patients (10). As a result, adenomyosis pain elimination is a crucial clinical concern.

Pain is commonly associated with inflammation and injury (11). There is accumulating evidence that immune cells interact with nociceptor sensory neurons during inflammation to regulate the levels of pain, host defense, and the progression of inflammation (11, 12). Immune cells around the peripheral nerve terminals release a variety of cytokines, lipids, proteases, and growth factors to elicit nociceptor neuron– expressed receptors, leading to pain sensitivity (11, 13, 14). However, an issue remains unresolved—where do the elicit nociceptor neurons in adenomyosis lesions come from? Who is promoting the growth of the nerve terminals?

Despite the fact that the pathophysiology and molecular pathways of adenomyosis remain unsolved, several theories have been offered to account for the development of the disease, including metaplastic myometrial stem cells (15). This stem cell hypothesis has been divided into two scenarios: one in which the ectopic endometrium is thought to have developed *de novo* from embryonic epithelial progenitor cells (16) and another in which adult endometrial stem cells have been able to enter the myometrium and differentiated for unknown reasons (17, 18). The majority of current research, however, has focused on two distinct types of stem cells: endometrial epithelial progenitor cells and mesenchymal stem cells (eMSCs) (19). Little research has been conducted to determine if these stem cells can differentiate into neural progenitor cells or even neurons.

The advent of single-cell sequencing technology now solves this problem considerably that previous studies have focused limitedly on one or a couple of dysfunctional cells in adenomyosis, resulting in a lack of unbiased investigations to explore the impact of heterogeneous cells on the neural development of adenomyosis. Single-cell sequencing technology has gained widespread application in numerous fields (20, 21). In the present study, in order to explore the mechanisms of adenomyosis pain, we applied single-cell sequencing technology to comprehensively analyze the characteristics of cells within the lesions of adenomyosis. Here, we identified a novel type of NTK cells in the foci of patients with adenomyosis pain that regulates cell differentiation and even induces stem cells to differentiate into neural-like cells.

Natural killer cells (NKT cells) were identified as a distinct lymphocyte lineage defined by the expression of a single invariant T-cell receptor (TCR) chain encoded by V24J18 in humans, which is predominantly associated with a restricted repertoire of TCR chains, primarily V11 in humans (22–24). Type I and type II NKT cells have been identified as the subpopulations of NKT cells, and both of these subpopulations recognize CD1d-presented lipid antigens, respectively (25, 26). Despite the fact that NKT cells constitute a small fraction of lymphocytes, they play an essential role in the immunomodulation of a wide range of diseases, including infections, autoimmune diseases, tumors, and even neurologically associated disorders (27, 28). NKT cells are widely accepted to be capable of secreting significant amounts of cytokines such as interferon-gamma (IFN-gamma), interleukin-17 (IL-17), interleukin-4 (IL-4), and interleukin-10 (IL-10), all of which are required for the initiation and modulation of diverse immune responses (29), but it has been shown in research that invariant (type I) NKT cells may block Th17 differentiation in the absence of cytokines (30). It is unsurprising that the cytokines released by NKT cells are also capable of regulating cell differentiation in addition to inducing an immune response.

In the present study, we identified NKT cells in the foci of patients with adenomyosis pain by single-cell sequencing and deduced the relationship between novel NKT cells and neuronal cell differentiation at the adenomyosis lesion.

## Methods

### Sample collection and ethics

Tissue samples were collected from 10 adenomyosis patients with dysmenorrhea (AM_Pain group) [visual analog scale (VAS) ≥4] and 12 adenomyosis patients without dysmenorrhea (AM_Painfree group) as controls undergoing hysterectomy (details in **Table 1**). The MRI images of all patients indicated that the entire myometrium was affected by adenomyosis. The visual analog scale (VAS) was used to assess the level of dysmenorrhea. The left end of the VAS was scored as 0 to represent “no pain,” while the right end was scored as 10, meaning the “most severe pain imagined” (31, 32). All enrolled patients did not receive hormone treatment in 6 months. Among them, the tissues from two adenomyosis patients with painful menstruation and two control patients were subjected to single cell RNA-sequences (scRNA-seq) and the other 36 samples were utilized for the validation using quantitative real-time polymerase chain reaction (qRT-PCR) or multiplex immunofluorescence. This project was approved by Ethics Committee of Ningbo Women and Children’s Hospital (NO. EC2022-M012).

**Table 1:** Subject's Characteristics in women with and without AM pain.

Parameters	AM painfree (n=10)	AM pain (n=8)	P value
**Age**	38±5.9	37±4.17	0.6913
**Menstrual cycle phase**			
Proliferative	3(30%)	3(37.5%)	
Secretory	7(70%)	5(62.5%)	
**Pain-related information**			
***Duration of chronic pain(y)***
≤2 years		1(12.5%)	
>2 years ≤5 years		4(50%)	
>5 years		3(37.5%)	
***VSA score***			
>4 ≤6		3(37.5%)	
>6		5(62.5%)	
***Administration of pain medication***			
Yes		2(25%)	
NO		6(75%)	
**Size of Cyst(cm^3^)**			
<100cm^3^	4(40%)	3(37.5%)	
<500cm^3^, ≥100cm^3^	4(40%)	4(50%)	
≥500cm^3^	2(20%)	1(12.5%)	

### Cell preparation

Adenomyosis tissues were digested into a single-cell suspension by collagenase IV(17104019, Gibco, USA) in 37°C for 2 h. The undigested tissue pieces were removed by a 100 μm cell filter. Cells were collected with centrifuge and washed three times. Samples were next diluted with PBS (phosphate buffered saline) containing 0.04% Bovine Serum Albumin (BSA, BBI, Shanghai, China) to the density of approximately 1 × 10^6^ cells/ml. The density of live cells was counted by an automated cell counter (Thermo Fisher, USA). The cell viability of samples used for scRNA sequencing was 92% for adenomyosis with dysmenorrhea sample and 91% for the control sample.

### scRNA-seq using 10×genomics

The density of the single-cell suspension was adjusted to 1,100 cells/μl. The cell suspension was mixed with gel beads including barcodes and the reaction solution. The mixture was loaded into chromium chip B *via* the microfluidic “double-cross” system to the formation of gel bead in emulsion (GEM). The remaining procedures including reverse transcription and the library construction were performed according to the standard manufacturer’s instructions. The DNA library was sequenced on NovaSeq with approximately 50,000–100,000 reads per cells. Single-cell analyses were performed using the lC-bio cloud platform. For quality control, low-quality cells (<3 cells/gene, <200 cells/gene, >6,500 genes/cell, >5% hemoglobin genes, and >30% mitochondrial genes) were removed. The number of reads, valid barcodes, sequencing saturation, and the Q30 base in Unique Molecular Identifiers (UMI) were similar among the four samples.

### Identification of cell cluster by uniform manifold approximation and projection analysis of scRNA-seq datasets

To identify major cell types, we performed uniform manifold approximation and projection (UMAP) using the Serat package of R software. To distinguish the cell types, we screened the marker genes through web-based CellMarker databases (http://biocc.hrbmu.edu.cn/CellMarker/). The detailed marker genes were listed below: IGHG1, IGKC, and IGLC2 for B cells; ITGAM, CD1C, and ITGAX for dendritic cells; CD68, TLR2, and CD163 for macrophage cells; KIT, FCER2, and TPSB2 for mast cells; KLRF1, KLRD1, and TRDC for NK cells; TM4SF1, PECAM1, and CDH5 for endothelial cells; CD3G and CD3E for T cells; GYPA for red blood cells; MMP11, SFRP1, and CD24 for multipotent stem cells; CDH1, KRT18, and EPCAM for epithelial cells; and CCL2, TWIST1, and ZEB2 for mesenchymal cells (more details in **Supplementary File 1**). The heat map and violin plots were generated from R package using the default complete-linkage clustering algorithm.

### Bioinformatics analysis of biological process enrichment, pathway enrichment prediction, and single-cell trajectories

We used the DAVID server to perform biological process enrichment analysis with significant differentially expressed genes in each cluster. We applied the gene sets of the Gene Ontology (GO) pathway to predict the downstream pathways of differential genes for each cell type. The monocle package of R software was used to analyze the single-cell trajectory in multipotent stem cells subtypes for discovering the developmental transition of multipotent stem cells.

### RNA extraction and quantitative real-time polymerase chain reaction

Total RNA was extracted from adenomyosis tissues using a TRIzol™ reagent (Life Technologies, Waltham, MA, USA), following the manufacturer’s instructions. Extracted RNA was resuspended in 20–30 μl RNase-free DEPC (diethypyrocarbonate) water and stored at −80°C. cDNA was synthesized using a transcription kit as per the manufacturer’s instructions (Vazyme Biotech, Nanjing, China). qRT-PCR analysis was conducted using the ChamQ SYBR qPCR Master Mix (Vazyme Biotech, Nanjing, China) on an Applied Biosystems 7500 Real-Time PCR System and associated software (Applied Biosystems, Waltham, MA, USA). After the initial denaturation step at 95°C for 15 s, three-step amplification was performed (95°C for 10 s, 60°C for 35 s) for 40 cycles. The relative expression levels of mRNAs were calculated with 2–ΔCt. GAPDH (glyceraldehyde-3-phosphate dehydrogenase) was used as an internal control. The specific mRNA primers used for qRT-PCR are presented in **Table 2**.

**Table 2:** Primer sequences.

Primer Name	Sequence
NEFM homo	F: 5’- AGGCCCTGACAGCCATTAC - 3’ R: 5’- CTCTTCGGCTTGGTCTGACTT - 3’
IGBP6 homo	F: 5’- GAGGGGCTCAAACACTCTACG - 3’ R: 5’- CCATCCGATCCACACACCA - 3’
GATA2 homo	F: 5’ - ACTGACGGAGAGCATGAAGAT - 3’ R: 5’ - CCGGCACATAGGAGGGGTA - 3’
HMGA1 homo	F: 5’ - GCTGGTAGGGAGTCAGAAGGA -3’ R: 5’ - TGGTGGTTTTCCGGGTCTTG - 3’

### Multiple immunofluorescence staining

Formalin-fixed paraffin-embedded adenomyosis tissues were sectioned into 4-μM-thick tissue slices. Slices were deparaffinized in xylene, rehydrated through graded ethanol, and boiled for 10 min in a citrate buffer (pH 6.0) for antigen retrieval. Endogenous peroxidase activity was suppressed by exposure to 3% hydrogen peroxide for 10 min at RT (room temperature). Tissue sections were then permeabilized by 0.1% TritonX-100 (Sigma-Aldrich, Darmstadt, Germany) for 10 min. Unspecific bindings were blocked by using PBS + 5% normal goat serum for 1 h at RT. Tissue sections were incubated overnight at 4°C with the following anti-IGFBP5(1:200, CST, Danvers, Massachusetts, USA), anti-PGP5.5 (1:200, abcam, Danvers, Massachusetts, USA), anti-CD8 APC (1:250, thermo, Waltham, MA, USA), anti-NKG7 (1:200, affinity, Suzhou, China), and anti-SFRP4 (1:200, abcam, Waltham, MA, USA). On the second day, slides were washed three times in PBS and incubated with the secondary antibody: goat anti-rabbit antibody 488 (1:500, abcam, Waltham, MA, USA), anti-mouse antibody 564(1:500, abcam, Waltham, MA, USA) and anti-rat antibody 405(1:500, abcam, Waltham, MA, USA) for 1 h at RT in the dark. Finally, sections were mounted using a fluorescent mounting medium and visualized under a fluorescent microscope (Leica, Germany) and a confocal laser scanning microscope (FV3000, Olympus, Japan).

### Statistical analysis

Statistical analysis was performed using GraphPad Prism version 8.0 (GraphPad) and SPSS software (version 20.0; IBM Corp., Armonk, NY, United States). Comparisons between two group experiments were performed using a two-tailed Student’s t-test, while multiple comparisons between the groups were analyzed using a one-way analysis of variance (ANOVA), followed by the Student–Newman–Keuls test. All experiments were performed in triplicate, and the quantification of results was presented as the mean ± standard deviation. p < 0.05 was considered statistically significant.

## Results

### Single-cell analysis of ectopic lesion of Adenomyosis with pain and pain-free

**Figure 1:**
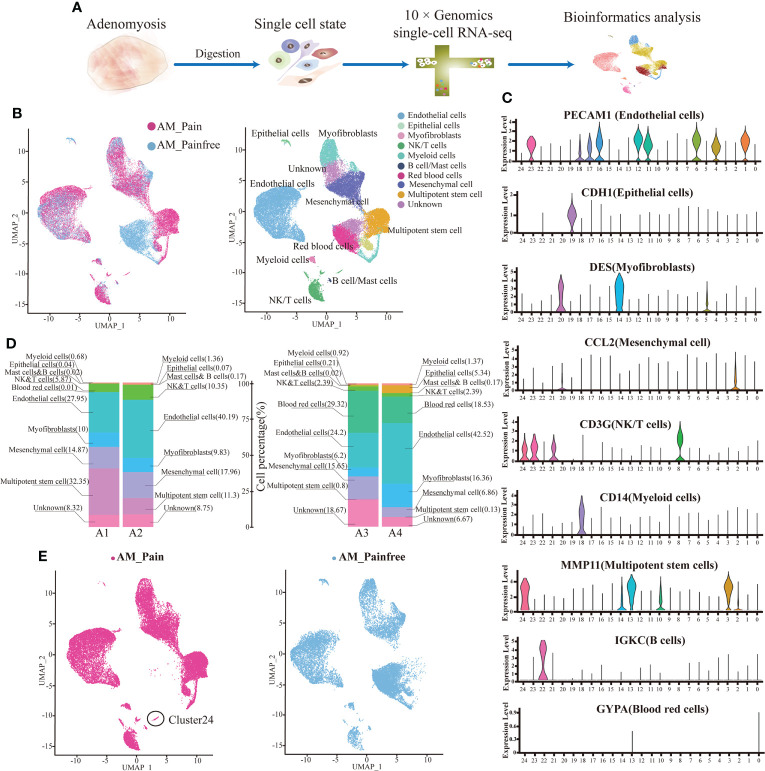
Identification of adenomyosis populations with single-cell transcriptomic analysis. **(A)** The workflow shows the collection and processing of obtained endometrium samples of AM_Painfree and AM_Pain for scRNA-seq. **(B)** Uniform manifold approximation and projection visualization of all cells displayed with different colors for samples. **(C)** For violin plots, x-axes stand for the numbers of clusters, and y-axes stand for the relative expression level of corresponding genes. **(D)** Percentage of the number of cells of each type. **(E)** Difference in clusters between AM_Pain and AM_Painfree groups. AM, adenomyosis.

To understand the potential molecular mechanisms of the hypersensitivity of neuroreceptors of AM, we analyzed lesion samples by scRNA-seq (**Figure 1A**). The scRNA-seq data were analyzed after initial quality control (QC) for filtering cells. A total of 15,658.5 ± 383.959 cells from AM-pain and 12,266 ± 4,414.761 cells from the AM-pain free group were sequenced and an average of approximately 32,332 reads per cell (**Supplementary File 2**). The cell transcriptomes from the four samples were combined and analyzed to detect the presence of potentially rare cell subtypes. UMAP analysis was performed, and 24 cell clusters were obtained. All of the cell clusters from the AM_Pain group and AM_Painfree group are shown in **Figure 1B**. The top differentially expressed genes of each cluster are shown in the heat map (**Supplementary File 3**). The 24 clusters were identified into 10 different cell types according to well-known marker genes (**Supplementary File 4**), including macrophages, dendritic cells, NK/T cells, epithelial cells, mesenchymal cells, myofibroblasts, B cells, mast cells, endothelial cells, red blood cells, and multipotent stem cells (**Figures 1B, C**). The proportion of each cell type in the tissue is shown in **Figure 1D**. An issue of interest is that the proportion of multipotent stem cells (including cluster 3 and 13) in the AM_Pain group was 30% compared to only 1% in the AM_Painfree group (**Figure 1D**). Additionally, cluster 24 was a unique cell group in the AM_Pain group (**Figure 1E**).

### SFRP4^+^ natural killer T cells were explicitly present in the tissues of patients with adenomyosis pain

**Figure 2:**
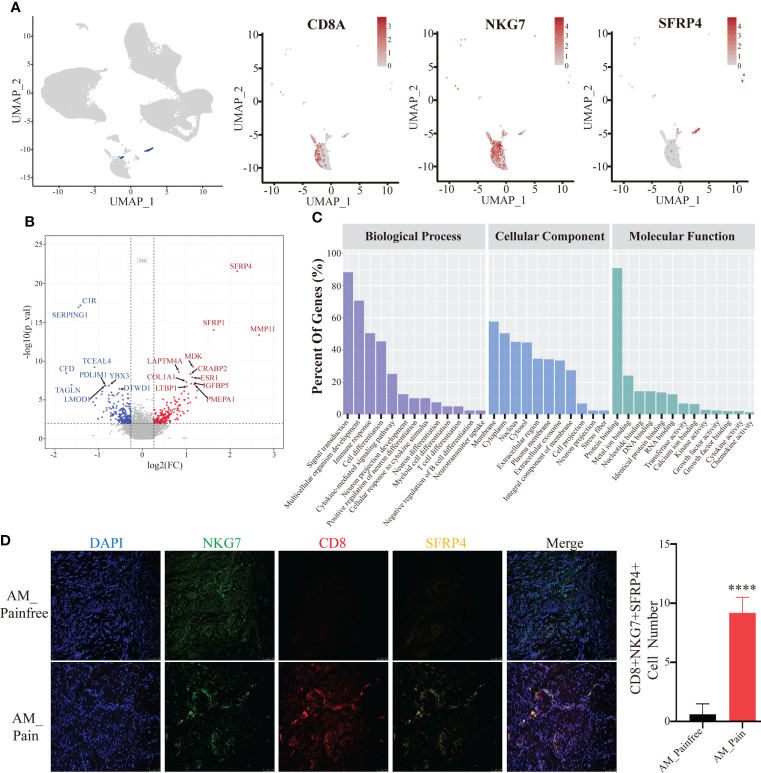
SFRP4^+^ NKT cells are specifically present in the AM_Pain group. **(A)** CD8, NKG7, and SFRP4 define the cluster 24 cell population. **(B)** Comparison of differential genes in cluster 24 cell groups (AM_Painfree group *vs*. AM_Pain group). **(C)** GO analysis of cluster 24 cell populations. **(D)** SFRP4-specific expression in NKT cells in the AM_Pain group.****<0.0001.

We discovered that cluster 24 was specifically present in the foci of patients with adenomyosis pain compared to the pain-free group by attributing the composition of cell subpopulations in the two groups. We screened cluster 24 for marker genes and observed that this cell population was ubiquitously expressed in the marker genes of natural killer T cells (NKT cells), CD8A, NKG7 (**Figure 2A**). We performed differential gene analysis on cluster 24 cell populations from both groups and found that the differential genes that were remarkably overexpressed in the adenomyosis pain group were *SFRP4* and *MMP11* (log2(FC)>2). In addition, *MDK, CRABP2, ESR1. IGFBP5*, and *PEMPA1* were presented high expression in the adenomyosis pain group (log2(FC)>1)(**Figure 2B**). The GO enrichment analysis of these differential genes indicates that they could influence cell differentiation and development, including neuronal and immune cells, *via* protein binding and might even affect neurotransmitter transmission in biological processes (**Figure 2C**). The SFRP family proteins have been widely documented to suppress cell differentiation *via* inhibiting the Wnt/-catenin signaling pathway (33, 34). This might explain the presence of large numbers of undifferentiated cells in the adenomyosis pain group. We used immunofluorescence and flow cytometry to clarify that SFRP4^+^NKT cells are abundantly present in the tissues of the AM_Pain group (**Figure 2D**).

### Multipotential stem cells possess the potential to differentiate into neural progenitor cells

**Figure 3:**
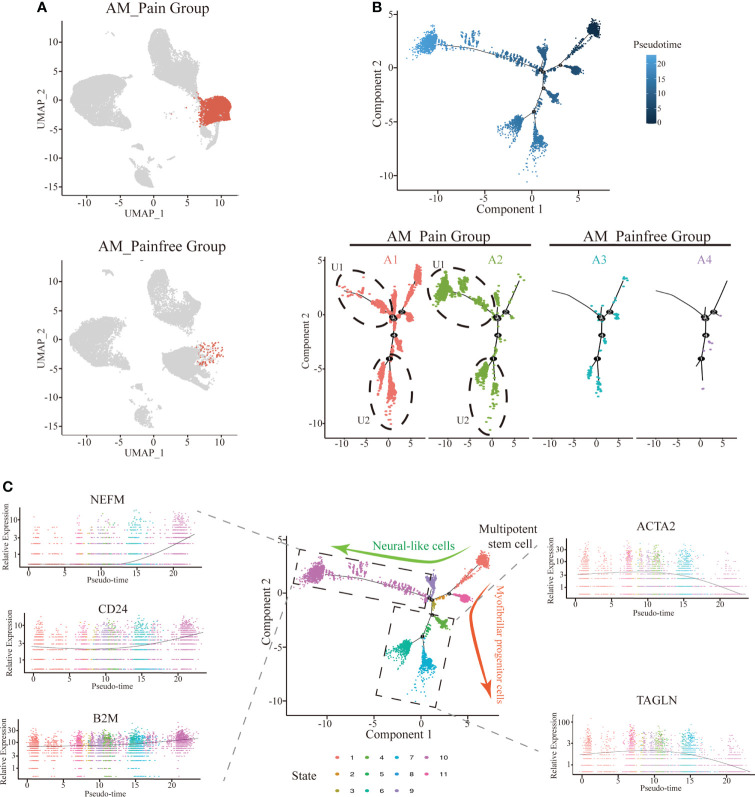
Differentiation of multipotent stem cells into neural-like cells in the single-cell trajectory analysis. **(A)** Comparison of cluster 3 and 13 cell numbers between AM_Painfree and AM_Pain groups. **(B)** Multipotent stem cells differentiated into two main cell populations in the AM_Pain group with the pseudotimeline, U1 and U2. **(C)** The U1 region cells mainly expressed nerve-associated markers, NEFM, CD24, and B2M, while the U2 region cells primarily expressed smooth muscle cell–associated markers, ACTA2 and TAGLN.

Because of the aberrant expression of SFRP4, we found that the number of cells in the multipotent stem cell population (i.e., cluster 3 and cluster 13) was substantially higher in the AM_Pain group compared to the AM_Painfree group (**Figure 3A**). In order to explore into what type of cells this large population of multipotential stem cells would eventually differentiate into, we performed the single-cell trajectory analysis on this cell population. The results of the pseudotimeline analysis implied that, as time progressed, there were statistically significant differences between the AM_Pain group compared to the AM_Painfree group in two directions, namely, the U1 cell population and the U2 cell population (**Figure 3B**). We identified two unknown cell populations of cell markers and discovered that the developmental direction of U1 progressively overexpressed NEFM, CD24, and B2M, which were associated with neural progenitors over time, while the developmental direction of U2 progressively overexpressed ACTA2 and TAGLN, which were related to smooth muscle cells (**Figure 3C**). Therefore, we assume that this cluster of stem-like multipotent progenitor cells has the potential to differentiate into neural progenitor cells.

### SFRP4^+^IGFBP5^hi^ NKT cells might be a crucial factor in the induction of neural-like cell differentiation

**Figure 4:**
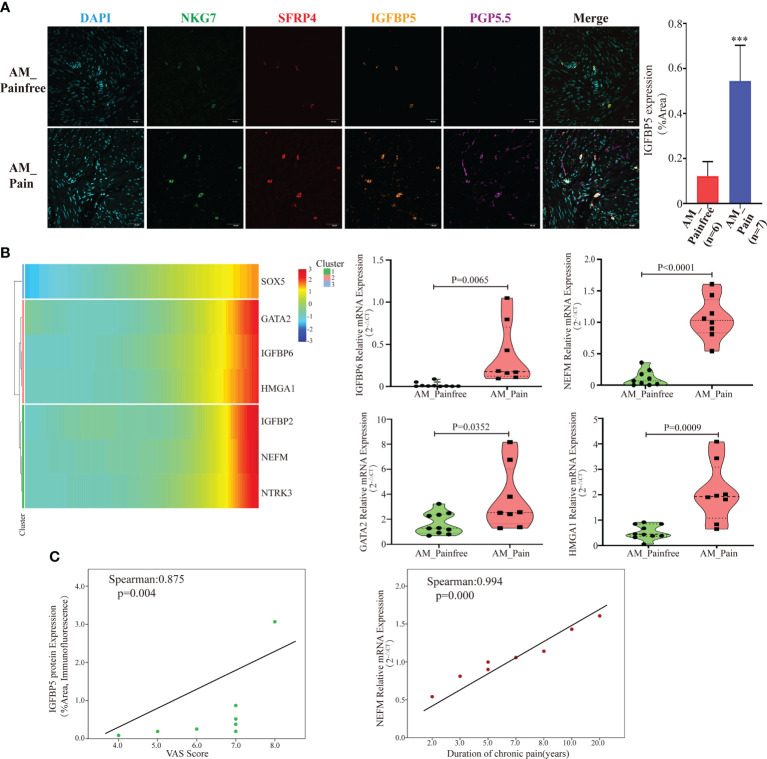
SFRP4^+^IGFBP5^hi^NKT cells induced neural-like cell differentiation and pain-related factor expression. **(A)** Immunofluorescence revealed a considerable number of nerve fibers in the AM pain group, as well as elevated IGFBP5 production by SFRP4^+^NKT cells. PGP5.5 stands for nerve fibers. NKG7 and SFRP4 stand for NKT cells. **(B)** Based on pseudotime analysis, a gene heat map displaying the expression of nerve development and pain-related components was under development. Validation of RT-PCR for associated mRNA expression in AM tissues. **(C)** The IGFBP5 protein expression level was correlated positively with the VAS score, while the NEFM mRNA expression level was correlated positively with the duration of discomfort. The data were distributed normally.

A further challenge is what enables these multipotent stem cells to ultimately differentiate into neural-like cells. According to the previous genetic analysis of cluster 24, it was observed that IGFBP5 was highly expressed in the AM_Pain group. The gene *IGFBP5*, which has been linked to neuropathic differentiation (35). Therefore, we suspected IGFBP5 might play a vital role in the process of multipotent stem cells differentiating into neurogenic cells. To investigate the origin of IGFBP5, we simultaneously labeled SFRP4^+^NKT cells, IGFBP5 protein, and nerve fibers (PGP5.5) by multicolor immunofluorescence. The immunofluorescence results indicated that the number of nerve fibers was significantly increased in the AM pain group compared to the AM non-pain group. Meanwhile, SFRP4^+^NKT cells were highly IGFBP5-expressing and secreted IGFBP5 was expressed in and around the nerve fibers themselves. (**Figure 4A**). Furthermore, the genetic heat map of the single-cell trajectory indicated that several genes related to nerve growth and neuroinflammatory factors, such as IGFBP6, NEFM, GATA2 and HMGA1, appeared to be highly expressed in multipotent stem cells at the end stage of differentiation (**Figure 4B**). In contrast, IGFBP5 expression remained consistently high throughout the differentiation process (**Supplementary File 4**). We verified that these genes indeed appeared highly expressed in AM_Pain group by RT-PCR (**Figure 4B**). To clarify whether these genes were associated with the duration of pain and VAS scores, we performed a correlation analysis, and first we identified that there was a positive correlation between the protein expression intensity of IGFBP5 and VAS scores(spearman:0.875, p=0.004). More importantly, there was also a positive correlation between the gene expression of NEFM and the duration of pain in AM_Pain patients (spearman:0.994, p=0.000)(**Figure 4C**).

## Discussion

Adenomyosis is a sex hormone–dependent condition induced by increased inflammation, which has an influence on apoptosis and neurovascular development (36, 37). Although more advanced diagnostic methods permit the majority of women with adenomyosis to be recognized sooner, there is mounting evidence that the onset of adenomyosis has gotten more youth (7, 38). According to statistics, 30% of patients diagnosed with adenomyosis are young women who experience peritoneal pain, irregular uterine bleeding, and infertility (39). As a consequence, a conservative strategy targeted at maintaining or restoring fertility while simultaneously controlling clinical symptoms should be investigated. However, existing care approaches are unsuccessful in reducing adenomyosis pain. Therefore, understanding the processes underlying adenomyosis pain is crucial for designing more effective treatment regimens.

Although only two samples were sequenced in both the adenomyosis pain group and the adenomyosis non-pain group in this single-cell sequencing, laboratory tests were performed on each group (n = 10 for AM_Painfree group and n = 8 for AM_Pain group) to confirm the presence of NKT cells and the increase in neural progenitor cells in the adenomyosis pain group in order to increase the feasibility of the results.

In comparison to the adenomyosis non-pain group, the adenomyosis pain group had a greater proportion of undifferentiated cell populations, including cluster 3 and cluster 13, and, even within the NKT (cluster 24) cell population, there was still a high level of expression of progenitor-related genes such as SFRP1 and MDK4. This phenomenon might be characterized by excessive SFRP4 expression in the cluster 24 cell population. Secreted frizzled-related protein 4 (SFRP4) is a member of the SFRP family that acts as a soluble regulator of Wnt signaling (40). Various studies have shown that SFRP4 has the ability to modulate a variety of biological processes. SFRP4 has been demonstrated in several studies to have dual functions, not only increasing apoptosis and negatively regulating differentiation (41–43) but also modulating cardiomyocyte (44) and skin keratinocyte differentiation in a targeted way (45). IF confirmed that SFRP4^+^ NKT cells were present in the lesions of patients with adenomyosis pain and produced a significant quantity of SFRP4 into the extracellular tissue space. According to the GO analysis, it was noticeable that SFRP4^+^ NKT cells might govern the differentiation of a range of cells, including myofibroblasts and immune cells, which explained the existence of a significant number of undifferentiated progenitor cells in the lesions of the adenomyosis pain group.

Despite the identification of cell populations, we observed a substantial population of undifferentiated cells with a stem-like origin in the adenomyosis pain group, but this could not explain the incidence of adenomyosis pain. Therefore, we performed the single-cell trajectory analysis of cluster 3 and 13 in the adenomyosis pain group to investigate which cell type would develop in the pseudotimeline. It was shown that, over time, one of the populations would express neurogenic markers, NEFM, CD24, and B2M. We also did qPCR on the adenomyosis pain group and the non-pain group and discovered that several mRNAs associated with nerve growth and neuroinflammatory factors, NEFM, GATA2, HMGA1, and IGFBP6, were considerably more strongly expressed in the adenomyosis pain group than in the non-pain group. More notably, we discovered that the NEFM mRNA of these neurogenic markers in the adenomyosis pain group exhibited a positive association with the duration of pain. The NEFM (neruofilament medium) is the main component that makes up the neurofilament. The function of neurofilaments forming the axonal skeleton is to maintain the caliber of neurons and to participate in intracellular transport to axons and dendrites (46, 47). Not only is NEFM considered as a positive signal for the successful differentiation of mature neurons (48), but it has also been defined as one of the possible candidates contributing to the fear of pain (49).

More importantly, we have bioinformatically predicted a gene that might stimulate the differentiation of this population of stem cells into neural-like cells. During the process of cluster 3 and cluster 13 evolution, cells consistently overexpressed IGFBP5, a gene that was similarly aberrantly expressed in SFRP4^+^ NKT cells. Insulin-like growth factor binding proteins (IGFBPs) are carrier proteins for insulin-like growth factors and may modulate the activity of IGFs. IGFBPs have been previously demonstrated to influence the development of mesenchymal stem cells; for example, IGFBP2 regulates lipogenic cell differentiation. IGFBP5 is also a member of the IGFBP family of proteins. Yang (2019) reported that IGFBP5 not only induces differentiation of MSCs to dentinal cells but also, more surprisingly, induces the differentiation of stem cells from the dental pulp (DPSCs) into vascular and neural cells (50). We found a high expression of IGFBP5 in both nerve fibers labeled by neurogenic proteins and surrounding SFRP4^+^ NKT cells in tissue slides from the adenomyosis pain group, which indicates that the IGFBP5 that induces the differentiation of stem cell clusters into neurogenic cells might be derived from SFRP4^+^ NKT cells. In addition, it was well known that IGFBP5 not only induces cell differentiation (51) but may also modulate the hypersensitivity of nociceptive neurons in a peripheral nerve injury mouse model (52). In the present study, the protein expression levels of IGFBP5 were also found to be positively correlated with pain scores (VSA).

In the present study, we performed a comprehensive investigation of foci from patients with adenomyosis pain by single-cell sequencing and identified the existence of a high number of stem cells that can be turned into neurogenic cells, offering a new proof for the stem cell hypothesis of adenomyosis. Meanwhile, the recently found SFRP4^+^IGFBP5^hi^ NKT cells may provide a novel therapeutic target for controlling adenomyosis pain. We will continue our study on the mechanism of SFRP4^+^IGFBP5^hi^ NKT cell presence and provide a laboratory basis for neurogenic stem cell transformation. However, in the present experiment, although we confirmed the presence of a large number of undifferentiated cells in the adenomyosis pain group, there was no further evidence on the biological functions of SFRP4 and IGFBP5. The origin and future direction of the differentiation of this population of stem cells still need to be demonstrated in a great amount of follow-up assays, and these will be the focus of our future research.

## Conclusion

In the current investigation, we discovered a novel cluster of NKT cells, SFRP4^+^IGFBP5^hi^NKT cells, in the foci of patients with adenomyosis pain by single-cell sequencing. More critically, we observed that these SFRP4^+^IGFBP5^hi^NKT cells were connected with a substantial number of undifferentiated stem cells in the foci. Furthermore, this cluster of cells was capable of converting part of the stem cells into neurogenic cells and inducing pain.

## Data availability statement

The data presented in the study are deposited in the GEO databease, accession number GSE218044.

## Ethics statement

The studies involving human participants were reviewed and approved by the ethical committee of the Ethics Committee of Ningbo Women and Children Hospital (NO. EC2022-M012). The patients/participants provided their written informed consent to participate in this study.

## Author contributions

YC and JingZ: Designed the study, conducted experiments, analyzed the data, performed the statistical analysis, and wrote the manuscript. JuZ and YS: Conducted experiments and analyzed the data. LC: Provided scientific support and discussion. QW: Critically reviewed the manuscript and provided scientific support for experiments. All authors read and approved the final version of the manuscript.

## Funding

This work is supported by a Project supported by the National Science Foundation for Young Scientists of China (Grant No. 81901459), Public Welfare Projects in Zhejiang Province (Grant No. LGF19H040003) and First Municipal Medical and Health Brand Foundation of Ningbo(Grant No. PPXK2018-06). Ningbo Natural Science Foundation Project(Grant No. 2022JCGY010631)

## Conflict of interest

The authors declare that the research was conducted in the absence of any commercial or financial relationships that could be construed as a potential conflict of interest.

## Publisher’s note

All claims expressed in this article are solely those of the authors and do not necessarily represent those of their affiliated organizations, or those of the publisher, the editors and the reviewers. Any product that may be evaluated in this article, or claim that may be made by its manufacturer, is not guaranteed or endorsed by the publisher.
