# Supplementary figures and images for: SFRP4+IGFBP5hi NKT cells induced neural-like cell differentiation to contribute to adenomyosis pain
Source: Front Immunol. 2022 Nov 30;13:945504. doi: 10.3389/fimmu.2022.945504 (PMC9750790; doi:10.3389/fimmu.2022.945504)

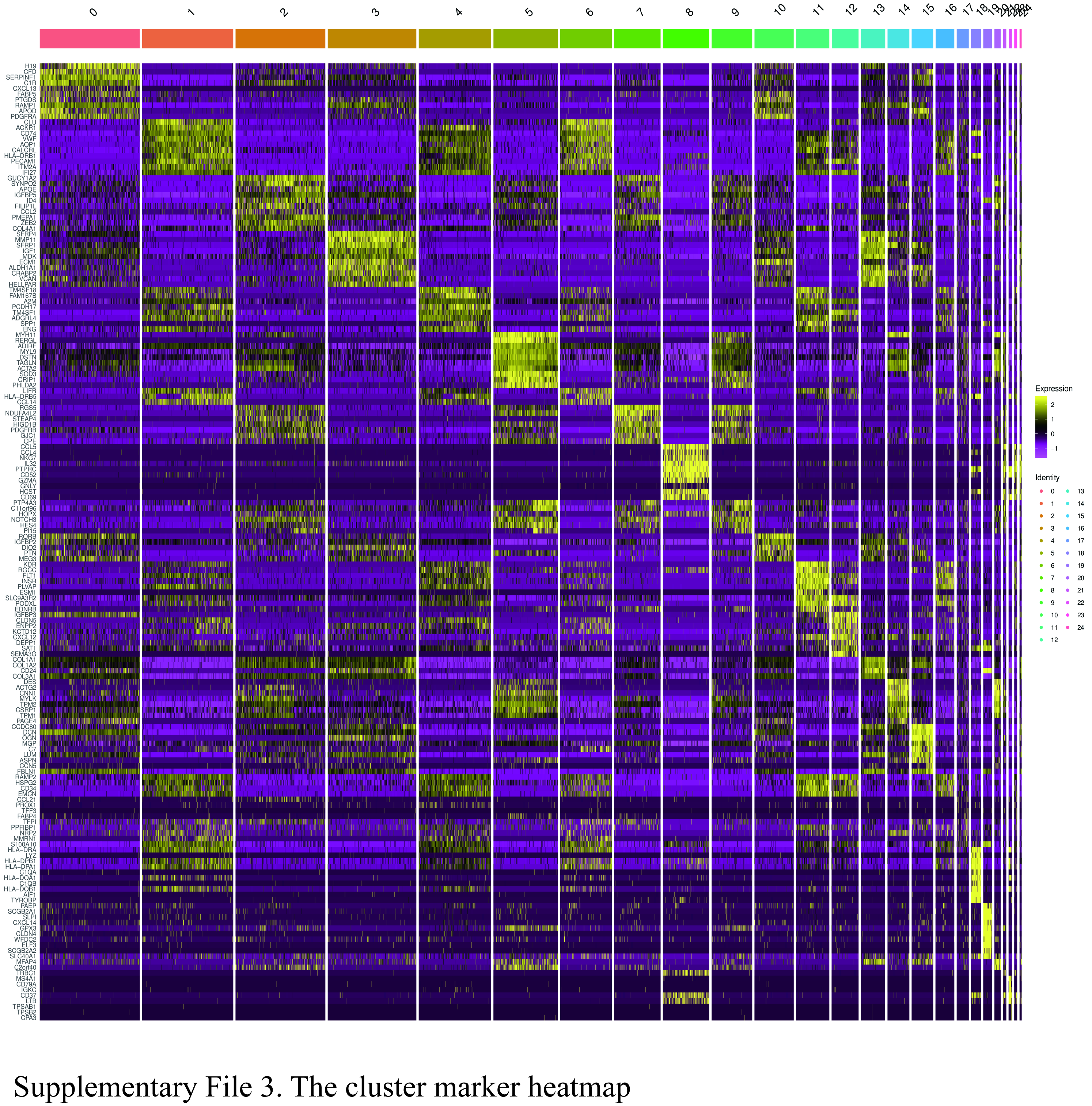

Supplement: Supplementary file 1 [file Image_1.tif]

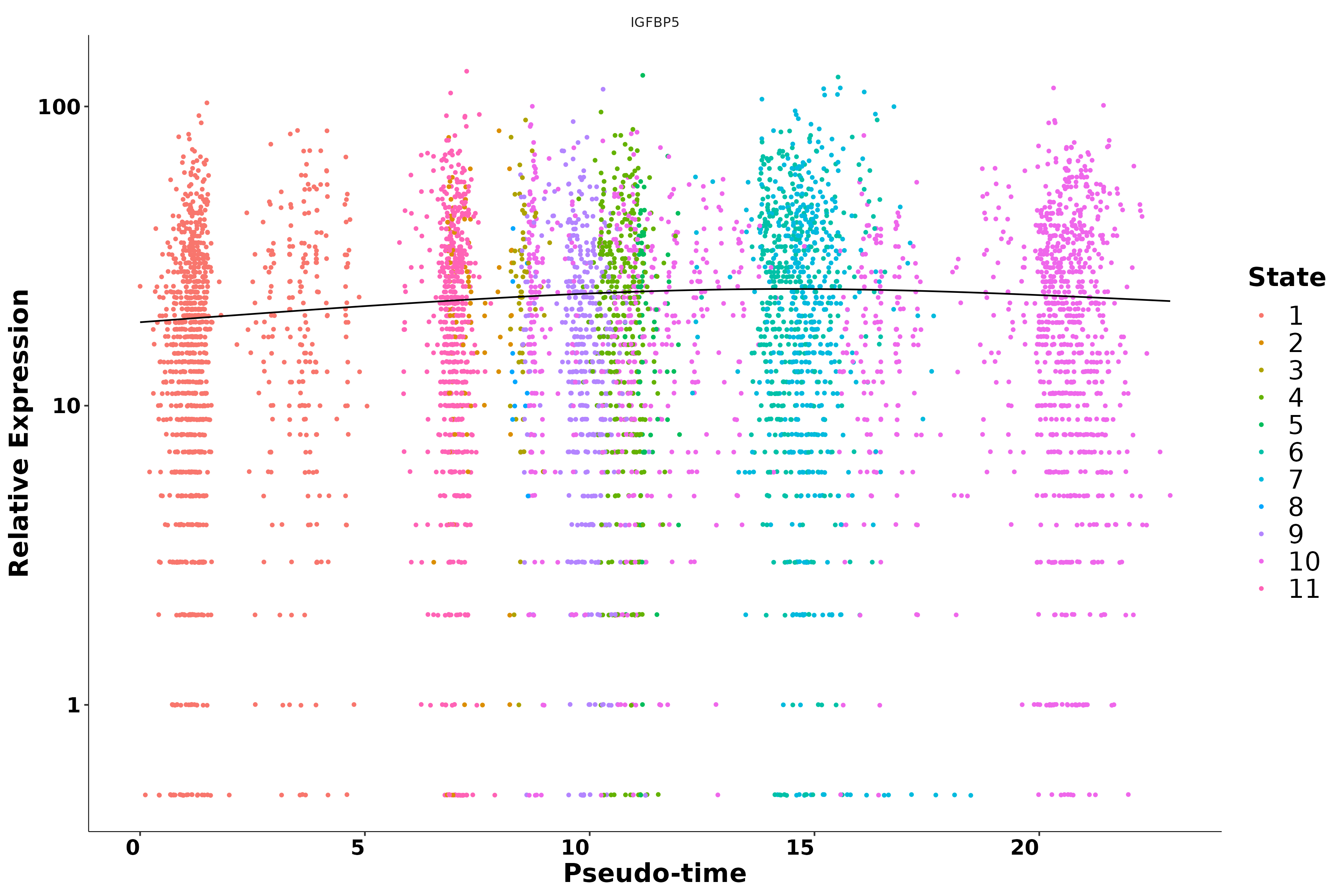

Supplement: Supplementary file 2 [file Image_2.tiff]

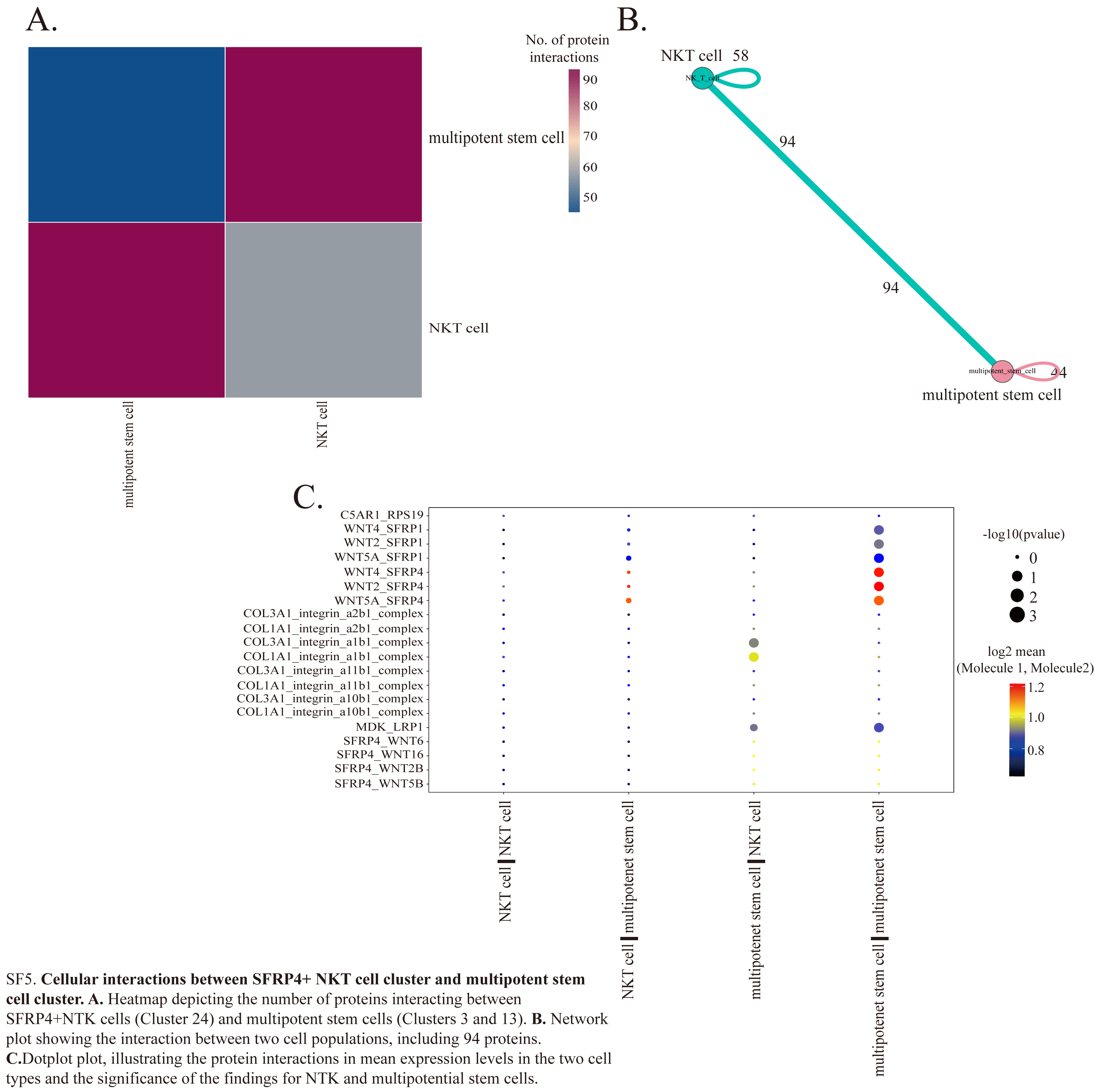

Supplement: Supplementary file 3 [file Image_3.tif]
