# Supplementary material for: SFRP4+IGFBP5hi NKT cells induced neural-like cell differentiation to contribute to adenomyosis pain
Source: Front Immunol. 2022 Nov 30;13:945504. doi: 10.3389/fimmu.2022.945504 (PMC9750790; doi:10.3389/fimmu.2022.945504)
Supplement: Supplementary file 4 [file Table_1.docx]

**Supplementary file1. The identification of cell markers.**

| Macrophages | Mast cells | NK cells | Endothelial cells | T cells | Red blood cells | Multipotent stem cell | Mesenchymal cell | Epithelial cells |
| --- | --- | --- | --- | --- | --- | --- | --- | --- |
| MRC1 | KIT | KLRD1 | TM4SF1 | CD3G | GYPA | MMP11 | CCL2 | CDH1 |
| CD68 | FCER2 | KLRF1 | PECAM1 | CD3E |  | SFRP1 | TWIST1 | KRT18 |
| TLR2 | TPSB2 | TRDC | CDH5 |  |  | CD24 | ZEB2 | KTR8 |
| CD163 |  | CD160 | VWF |  |  |  |  | EPCAM |
