# Supplementary material for: SFRP4+IGFBP5hi NKT cells induced neural-like cell differentiation to contribute to adenomyosis pain
Source: Front Immunol. 2022 Nov 30;13:945504. doi: 10.3389/fimmu.2022.945504 (PMC9750790; doi:10.3389/fimmu.2022.945504)
Supplement: Supplementary file 5 [file Table_2.docx]

| Sample | Estimated Number  of Cells | Mean Reads  per Cell | Median Genes  per Cell | Reads Mapped  to Genome | Reads Mapped  Confidently to Genome | Fraction  Reads in Cells | Total Genes  Detected |
| --- | --- | --- | --- | --- | --- | --- | --- |
| A2 | 15,930 | 26,016 | 1,877 | 97.4% | 91.4% | 88.3% | 35,379 |
| A3 | 13,407 | 30,550 | 2,224 | 96.7% | 91.1% | 91.5% | 37,035 |
| A4 | 9,145 | 47,529 | 2,554 | 97.5% | 90.5% | 88.5% | 34,731 |
| A_1 | 15,387 | 25,233 | 1,953 | 97.4% | 88.4% | 87.8% | 36,072 |

Supplementary file 2. The initial quality control (QC) for filtering cells
